# Providing early indication of regional anomalies in COVID-19 case counts in England using search engine queries

**DOI:** 10.1038/s41598-022-06340-2

**Published:** 2022-02-11

**Authors:** Elad Yom-Tov, Vasileios Lampos, Thomas Inns, Ingemar J. Cox, Michael Edelstein

**Affiliations:** 1 Microsoft Research, Herzliya, Israel; 2 Faculty of Industrial Engineering and Management, Technion, Haifa, Israel; 3 Department of Computer Science, University College London, London, UK; 4 Department of Computer Science, University of Copenhagen, Copenhagen, Denmark; 5 Azrieli Faculty of Medicine, Bar-Ilan University, Safed, Israel; 6 UK Health Security Agency, London, UK; 7 St Helens and Knowsley Teaching Hospitals NHS Trust, Merseyside, UK

**Keywords:** Signs and symptoms, Information technology, Infectious diseases

## Abstract

Prior work has shown the utility of using Internet searches to track the incidence of different respiratory illnesses. Similarly, people who suffer from COVID-19 may query for their symptoms prior to accessing the medical system (or in lieu of it). To assist in the UK government’s response to the COVID-19 pandemic we analyzed searches for relevant symptoms on the Bing web search engine from users in England to identify areas of the country where unexpected rises in relevant symptom searches occurred. These were reported weekly to the UK Health Security Agency to assist in their monitoring of the pandemic. Our analysis shows that searches for “fever” and “cough” were the most correlated with future case counts during the initial stages of the pandemic, with searches preceding case counts by up to 21 days. Unexpected rises in search patterns were predictive of anomalous rises in future case counts within a week, reaching an Area Under Curve of 0.82 during the initial phase of the pandemic, and later reducing due to changes in symptom presentation. Thus, analysis of regional searches for symptoms can provide an early indicator (of more than one week) of increases in COVID-19 case counts.

## Introduction

COVID-19 was first reported in England in late January 2020^1^. By the end of 2020, over 2.6 million cases and 75 thousand deaths were reported.

In early March 2020, the UK’s Health Security Agency (UKHSA; formerly Public Health England), University College London (UCL) and Microsoft began investigating the possibility of using Bing web search data to detect areas where disease incidence might be increasing faster than expected, so as to assist UKHSA in the early detection of local clusters and better planning of their response. Here we report on the results of this work, which provides UKHSA with weekly reporting on indications of regional anomalies of COVID-19.

Internet data in general and search data in particular, have long been used to track Influenza-Like Illness (ILI)^2–4^, norovirus^5^, respiratory syncytial virus (RSV)^6^, and dengue fever^7^ in the community. The added value of these data relies on the fact that most people with, for example, ILI will not seek healthcare but will search about the condition or mention it in social media postings^8^. Such behaviour could be compounded by fear of attending medical facilities in the midst of a pandemic. This enables the detection of health events in the community before they are reported by formal public health surveillance systems and sometimes even when those events are not visible to the health system. Early evidence suggests that people with COVID-19 search the web for relevant symptoms, making such searches predictive of COVID-19^9^.

Building on these studies we aimed to identify local areas in England (specifically, Upper Tier Local Areas, UTLAs) with higher than expected rises in searches for COVID-19 related terms, in order to provide local public health services with early intelligence to support local action. We focused on the regional level because much of the response to the pandemic was coordinated at this level, and also because detecting local clusters while they may still be undetected by national level surveillance and before they have spread further is an efficient approach to outbreak management.

## Results

Our results below show that symptom searches were correlated with case counts, and that our approach allowed the prediction of regional anomalies approximately 7–10 days before they were identified using case counts during the first stages of the pandemic.

### Prediction quality for case number using geographically proximate UTLAs

The correlation between case counts of pairs of geographically proximate UTLAs which were at least 50km apart was, on average, 0.84. This is compared to 0.63 for randomly selected UTLA pairs (sign test, $P<10^{-9}$). Thus, as described in the “Methods” section, here we define anomalies as rises in one UTLA which are not observed in a nearby UTLA. As proximate UTLAs have correlated case counts, such mismatches are indicative of an anomaly at the UTLA level.

### Correlation of individual keywords with case counts

**Figure 1:**
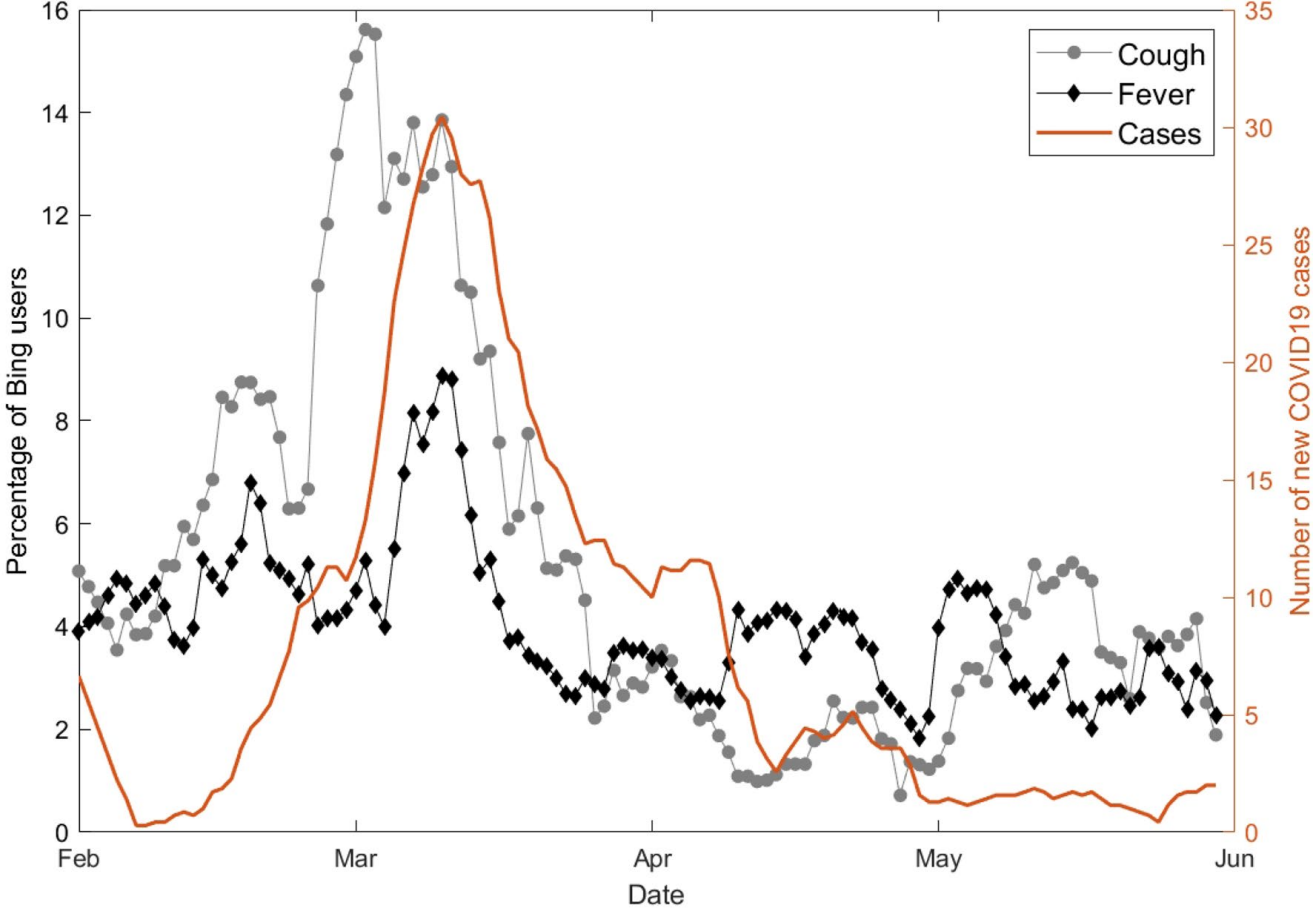
Number of COVID-19 cases (brown) and percentage of Bing users who queried for “cough” in a sample UTLA (gray circles) and for “fever” (black deltoids). Curves are smoothed using a moving average filter of length 7.

For illustrative purposes, Fig. 1 shows the daily number of COVID-19 cases and percentage of Bing users who queried for “cough” and “fever” in one of the UTLAs during the first wave of the pandemic. We calculated the cross-correlation between the daily time series of query frequencies for each keyword and the daily case count for each UTLA. The highest correlation and its lag in days were noted, and the median values (across UTLAs) are shown for each keyword in Table 1.

**Table 1:** Correlation (median across UTLAs) (corr) and lag (median across UTLAs)(in days) at which it is achieved, between case numbers and fraction of users who queried for these keywords on Bing.

Keyword	All periods		Period 1		Period 2		Period 3		Period 4	
Dates	1 Mar 2020–13 Dec 2021		1 Mar 2020–31 May 2020		1 Jun 2020–31 Aug 2020		1 Sep 2020–30 Apr 2021		1 May 2021–13 Dec 2021	
	Corr	Lag	Corr	Lag	Corr	Lag	Corr	Lag	Corr	Lag
Altered consciousness	0.034	0	0.125	0	0.137	0	0.066	0	0.060	0
Anorexia	0.066	19	0.173	9	0.191	10	**0**.**095**	8	0.086	10
Anosmia	0.040	11	0.278	8	0.191	11	**0**.**128**	5	0.108	6
Breathing difficulty	0.041	22	0.199	17	0.174	14	0.064	5	0.064	10
Chest pain	0.086	24	0.190	14	0.184	11	0.076	4	0.070	11
Chills	0.038	17	0.192	12	**0**.**201**	11	0.049	4	0.031	10
Cough	0.092	25	**0**.**383**	21	0.189	7	**0**.**113**	5	**0**.**152**	4
Diarrhea	0.138	26	0.172	14	0.191	13	0.049	5	0.083	11
Dry cough	0.011	17	0.272	18	0.186	8	0.090	8	0.114	8
Fatigue	0.091	23	0.154	2	0.174	12	0.015	2	0.074	15
Fever	0.048	28	0.289	17	0.160	8	0.052	10	0.076	24
Head ache	0.122	27	0.174	15	0.176	14	0.004	2	0.052	13
Joint ache	0.042	10	0.175	4	0.182	9	0.056	5	0.083	9
Muscle ache	0.059	18	0.174	9	**0**.**207**	8	0.031	4	0.074	9
Nasal congestion	0.092	22	0.191	13	0.195	10	0.073	10	0.091	9
Nausea	0.128	24	0.154	10	0.183	10	0.060	5	0.077	13
Nose bleed	0.061	20	0.153	13	0.186	10	0.052	6	0.065	7
Pneumonia	− 0.009	23	**0**.**319**	23	0.175	18	0.086	2	0.126	9
Rash	0.189	23	0.146	3	0.151	10	0.018	4	**0**.**187**	0
Runny nose	0.061	23	0.263	21	**0**.**212**	9	0.067	10	0.115	8
Seizure	0.103	26	0.12	12	0.20	10	0.017	7	0.057	16
Sneezing	0.016	25	0.155	18	0.161	7	0.076	5	0.088	9
Sore throat	0.055	26	**0**.**314**	20	0.197	9	0.084	12	**0**.**134**	7
Tiredness	0.066	24	0.140	18	0.177	11	0.019	5	0.057	15
Vomiting	0.111	25	0.149	12	0.201	9	0.051	4	0.089	7

A positive lag means that Bing searches appear before case counts, and vice versa. The three most strongly correlated keywords in each time period are highlighted. Synonyms are grouped into their respective main symptom.

**Figure 2:**
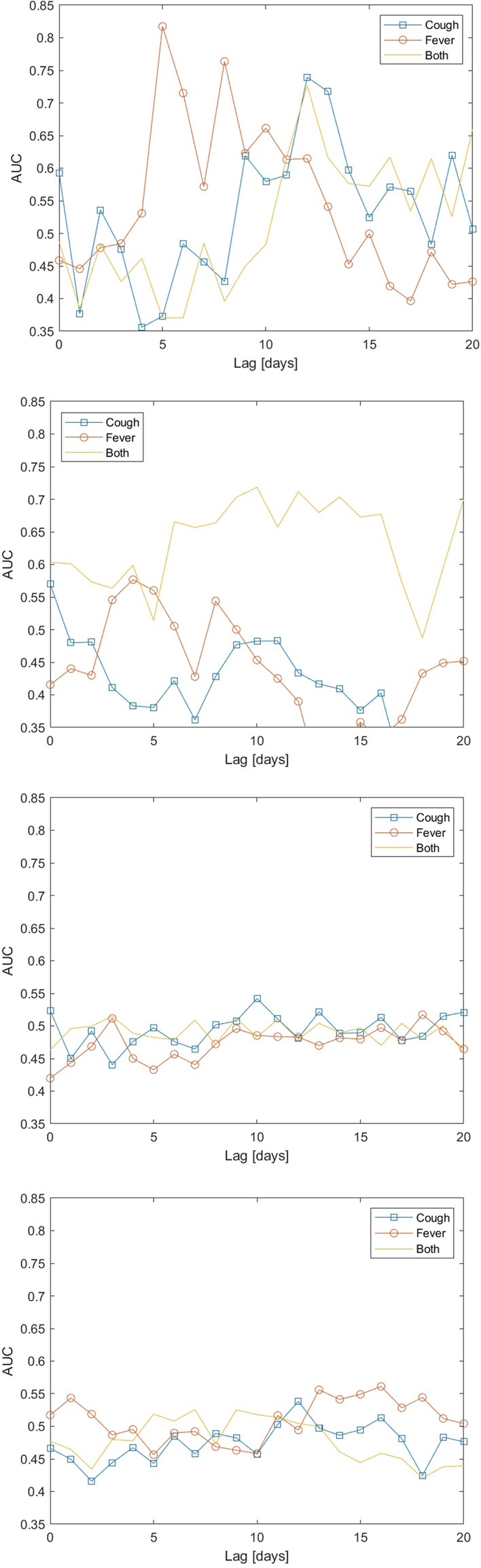
Area Under Curve (AUC) of the UTLA outlier measure for detecting unusually large rises in COVID-19 cases per UTLA, as a function of the lag between case counts and Bing data. The four figures refer to the four time periods: First wave (top) to fourth wave (bottom). Dates of the 4 periods are: (1) March 1st to May 31st. 2020, (2) June 1st to August 31st, 2020, (3) September 1st, 2020 to April 30th, 2021, and (4) May 1st, 2021 to December 13th, 2021. Curves are computed for all weeks and all UTLAs at each time period.

**Figure 3:**
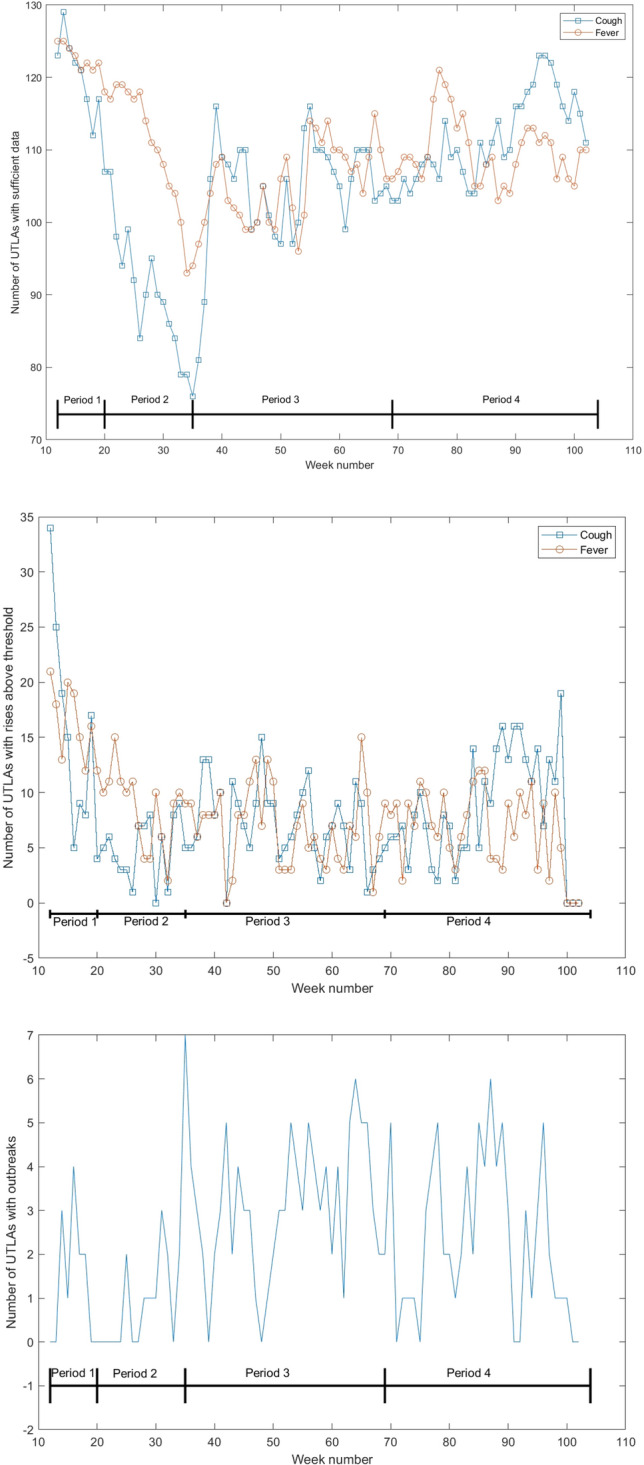
Number of UTLAs with sufficient Bing data over time (top), number of UTLAs with values over the threshold over time (middle) and number of UTLAs with an anomaly (as defined in the “Methods” section) (bottom). Week numbers correspond to the weeks since the beginning of 2020. The periods marked are: (1) March 1st to May 31st. 2020, (2) June 1st to August 31st, 2020, (3) September 1st, 2020 to April 30th, 2021, and (4) May 1st, 2021 to December 13th, 2021.

As the Table shows, the correlations and lag vary across the four periods ((1) March 1st to May 31st. 2020, (2) June 1st to August 31st, 2020, (3) September 1st, 2020 to April 30th, 2021, and (4) May 1st, 2021 to December 13th, 2021). During the first period, the best correlations at lags of up to 21 days were reached for ”cough”, ”sore throat”, and ”fever”. Based on initial results and using UKHSA case definition of COVID-19 at the time, we focused on two keywords, ”cough” and ”fever”, for the remaining analysis. We note, however, that more accurate results could have been achieved by tuning the model over time to account for the change in the most predictive symptoms.

### Detection ability of the outlier measure

We provided predictions for UTLAs where at least 10000 users queried on Bing in a one week interval. On average this corresponded to predictions for 106 UTLAs (of 173 UTLAs) per week.

Figure 2 shows the performance of the method over time by presenting the Area Under Curve (AUC, see Appendix for a sample ROC curve) where the dependent variable is the UTLA outlier measure calculated at different lags from the dependent variable. The latter is whether an actual outlier of cases was detected at a UTLA (see “Methods” section for details). As the Figure shows, performance changed over the duration of analysis. During the first wave of the pandemic, “fever” reached the highest AUC preceding case numbers by 5-8 days. During the second period of the pandemic the composite signal, calculated as the product of the UTLA outlier measure values for ”fever” and ”cough” (denoted in the figure as “Both”), reached the highest AUC at a slightly longer lead time (8-15 days), while during the third and fourth periods lags were longer still, but performance was overall lower than for the first two periods.

### Changes in detections over time

Figure 3 shows the number of UTLAs with sufficient data, meaning that enough users queried for the relevant terms, over the weeks of the analysis. As the figure shows, the number of users asking about “fever” and about “cough” were relatively high initially, but later had a significant drop (corresponding to the drop in cases), followed by a rise during the second wave of the pandemic. Figure 3 (center) shows the number of UTLAs per week that had rises above the threshold. Here too both “cough” and “fever” roughly follow the phases of the pandemic, meaning that more outbreaks were predicted during the first and third phases of the pandemic. Finally, the bottom figure shows the number of UTLAs which experienced an anomaly, as defined in the Methods, week over week. This figure demonstrates that the number of UTLAs which showed an anomaly was usually around 3 per week, with higher values observed in the 3rd and 4th periods of the pandemic.

### Demographic attributes of outlying areas

The 10 UTLAs that were false positives with the largest positive outlier measure and the 10 UTLAs that were false negatives with the largest negative outlier measure for “fever” at lags of 5 to 10 days at each week were identified to assess if they could by associated with specific demographic characteristics of their areas. Here, the highest correct detections were those where the largest predicted rise according to Bing data corresponded to a similar unexpected rise in case numbers and, similarly, incorrect detections were those where large predicted rises did not correspond to unexpected rises in case numbers.

Association between demographic characteristics of UTLAs and the the likelihood of incorrect detections was estimated using a logistic regression model. However, none of the variables were statistically significantly associated with these attributes ($P>0.05$ with Bonferroni correction) during the entire data period.

## Discussion

Internet data, especially search engine queries, have been used for tracking influenza-like illness and other illnesses for over a decade, because of the frequency at which people query for the symptoms of these illnesses and the fact that more people search for symptoms than visit a health provider^2,3^. COVID-19, which was a novel disease in early 2020, seemed to present similar opportunities for tracking using web data, and current indications suggest that search data could be used to track the disease^9^. However, COVID-19 also differs from diseases normally tracked using query frequencies, most commonly influenza. In particular, COVID-19 has disrupted daily life in ways that influenza does not and people with COVID-19 could need or be required to seek medical attention, thus making them more visible to the traditional healthcare system, e.g., general practitioners, medical clinics, and hospitals. Additionally, influenza has well documented seasonal activity, while COVID-19 activity has been prolonged. It was therefore unknown whether these different characteristics would affect online behaviour and as a consequence whether the methodological approaches used for other diseases would be appropriate for COVID-19. Furthermore, almost all previous work on disease surveillance using search data is based on supervised machine learning frameworks that rely on training data. However, there is little or no training data available for COVID-19. We therefore developed a method for detecting local outbreaks, based on past work^11,12^, that required minimal training data.

Our results demonstrate the highest correlation between case numbers and the use of the keywords “cough”, “fever” and “sore throat” at lead times up to 21 days, during the first period of the pandemic. Queries lead case numbers by 17-21 days (similar to the findings of^9^). Based on early indications of the apparent symptoms of COVID-19 from UKHSA we focused on using the first two keywords in our detection methodology.

The keywords most correlated with anomalies and with case counts changed over time, as has been observed for other conditions (e.g., influenza^13^). This suggests that the model would need to be adjusted over time to focus on the most relevant keywords.

The detected anomalies provided UKHSA with a lead time of approximately one week with respect to case numbers, initially with an AUC of approximately 0.82. This AUC later decreased to around 0.70 during the second phase of the pandemic, and to non-significant levels thereafter. This modest accuracy is nonetheless useful as long as exceedance of the 2 standard deviations threshold is not interpreted at face value as an increase in disease incidence, but as an early warning signal that triggers further investigation and supports outputs from other disease surveillance systems.

The results of this analytical method were integrated into routine UKHSA COVID-19 surveillance outputs together with a variety of other data sources. This type of information added value to the public health response as it was provided in a timely way, was flexible to potential changes in case definition and was complementary to other sources of syndromic surveillance at the early stages of infection before people seek healthcare. However, there were issues of completeness and representativeness of these data, alongside challenges of explaining model results to public health stakeholders.

The correlations between symptom search rates and case counts, even for the best performing keywords, were lower than correlations observed for other conditions (e.g., norovirus^5^ or RSV^6^). We hypothesize that this is due to several factors, including (i) data availability, (ii) changes in how people’s experience of the pandemic is manifested by searches online and public interest in the pandemic, which may have heightened awareness of the disease causing more people to query about it even if they did not experience symptoms, (iii) noisy ground truth data (i.e., case count data), which was strongly affected by testing policies and test availability, and (iv) attributes of the COVID-19 pandemic, which presents a more diverse set of symptoms, compared to, for example, influenza-like illness. We discuss each of these factors below:

Search data is noisy^14^ and Bing’s market share in England is estimated at around 5%^15^. The latter could be mitigated by using data from the dominant search engine (Google), though at the time of writing these data are not available for use by researchers or public health practitioners. Future work will test the hypothesis that data from a larger market share could have improved prediction accuracy.

User behavior may change over time (see, for example, Fig. 3). This can happen either as knowledge about COVID-19 improved or as a result of “COVID fatigue”, e.g., declining interest among people in addressing the pandemic. Moreover, different strains of the virus may cause different symptom profiles and anxiety among people, leading to different search behaviors. Mapping and understanding these changes is an important research question, which would enable adjustment of the model to improve its accuracy and public health utility.

A third factor affecting the reported performance is ground truth. We compared our results to the change in the number of positive COVID-19 cases. These numbers are affected by case definition and by testing policy, which may have caused a non-uniform difference between known and actual case numbers in different UTLAs. Additionally, COVID-19 has a relatively high asymptomatic rate (estimated at 40–45%^16^). People who do not experience symptoms would be less likely to be searching for these symptoms online and perhaps also missed in case number counts, though the extent of the latter is dependent on testing policy. On the other hand, serological surveys^17^ suggest that at the end of May 2020, between 5% and 17% of the population (depending on area in England) had been exposed to COVID-19, compared to only 0.3% that have tested positive to a screening test, suggesting that a large number of people who may have experienced symptoms of COVID-19 and queried for them were not later tested, leading to errors in our comparison between detections and known case numbers. We note that Virus Watch, a syndromic surveillance study^18^, and models based on Google search data^19^ also reported significant differences between their respective indicators and reported case numbers. Additionally, we report a specific outlier measure, which would not be sensitive, for example, if rises were to occur in a large number of UTLAs.

Finally, the COVID-19 pandemic is unique in its duration and for the rapid emergence of strains with slightly different clinical presentations^20^. This poses a unique challenge for detection based on internet data because it means that case identification changes, sometimes rapidly, meaning that models need to be adjusted over time. This is in contrast to diseases such as influenza, where symptoms are well established and are mostly stable over time. This presents a new and emerging challenge for scientists working in this area and reinforces the need for close collaboration between computer scientists, data scientists and epidemiologists, to ensure that case definitions are in line with the current epidemiology of the disease.

Despite these challenges to the accuracy of this model, the results were successfully integrated into routine UKHSA surveillance outputs and used for the surveillance of COVID-19. Future work should formally evaluate these outputs in the context of a public health surveillance system, to understand ways that the model results could be more effectively applied.

## Methods

Models of ILI which are based on internet data are usually trained using past season’s data. Since this was infeasible for COVID-19 we chose a different approach in our prediction, which utilized less training data. Our methodology examined two consecutive weeks, where during the first of those weeks we found, for each Upper Tier Local Authority (UTLA, a subnational administrative division of England into 173 areas^21^), other UTLAs with similar rates of queries for symptoms. These UTLAs were then utilized to predict the corresponding rates of queries for symptoms during the following week. A significant difference between the actual and predicted rate of searches served as an indication of an unusual number of searches in a given area, i.e., an anomaly.

This methodology is similar to prior work^11^, albeit one where differences are calculated between actual and predicted symptom rates. As such, it shares similarities with the methodology used to predict the effectiveness of childhood flu vaccinations using internet data^12,22^.

### Symptom list and area list

The list of 25 relevant symptoms for COVID-19 was extracted from UKHSA reports^10^, and are listed in Table 2 together with their synonyms, taken from Yom-Tov and Gabrilovich^23^.

In order to maximise the utility of the analysis, we conducted it at the level of the UTLA, over which local government has a public health remit.

**Table 2:** 25 symptoms related to COVID-19 (as identified by UKHSA^10^) and their synonyms or related expressions.

COVID-19 symptoms	Synonyms or related expressions
Altered consciousness	Altered consciousness
Anorexia	Appetite loss, loss of appetite, lost appetite
Anosmia	Loss of smell, can’t smell
Arthralgia	Joint ache, joint aching, joints ache, joints aching
Chest pain	Chest pain
Chills	Chills
Cough	Cough
Diarrhea	Diarrhea, diarrhoea
Dry cough	Dry cough
Dyspnea	Breathing difficult, short breath, shortness of breath
Epistaxis	Nose bleed, nose bleeding
Fatigue	Fatigue
Head ache	Head ache, headache
Myalgia	Muscle ache, muscular pain
Nasal congestion	Blocked nose, nasal congestion
Nausea	Nausea, nauseous
Pyrexia	Fever, high temperature
Pneumonia	Pneumonia, respiratory infection, respiratory symptoms
Rash	Rash
Rhinorrhea	Runny nose
Seizure	Seizure
Sore throat	Sore throat, throat pain
Sternutation	Sneeze, sneezing
Tiredness	Tiredness
Vomiting	Vomit, vomiting

### Search data

We extracted all queries submitted to the Bing search engine from users in England. Each query was mapped to a UTLA according to the postcode (derived from the IP address of the user) from which the user was querying. We counted the number of unique users per week who queried for each of the keywords within each UTLA, and normalized by the number of unique users who queried for any topic during that week within each UTLA. We counted users and not searches since a single user could generate multiple searches and counting users should correlate better with case counts. The fraction of users who queried for keyword *k* or its synonyms at week *w* in UTLA *i* is denoted by $F_{wk}^i$. Note that the fraction of users who queried for keyword *k* is the fraction of people who queried for keyword *k* *and its synonyms* listed in Table 2.

Data was extracted for the period between March 1st, 2020 to December 13th, 2021. The data period was divided into 4 segments, corresponding to the first wave of the pandemic (March 1st to May 31st. 2020), a middle period (June 1st to August 31st, 2020), the second wave of the pandemic (September 1st, 2020 to April 30th, 2021) and its third wave (May 1st, 2021 to December 13th, 2021).

For privacy reasons, UTLAs with fewer than 10,000 Bing users were removed from the analysis. Additionally, any keyword *k* which was queried by fewer than 10 users in a given week at a specific UTLA, *i*, was effectively removed from the analysis of that UTLA by setting $F_{wk}^i$ to zero (see also Fig. 3).

### Validation data

We compared our detection methodology (described below) to unusual changes in the number of reported COVID-19 cases per UTLA. COVID-19 case counts were accessed from the UK government’s coronavirus dashboard^24^. We used case counts as a proxy for disease incidence though this is known to be a noisy proxy (see Discussion).

Unusual changes in the number of cases were computed as follows: For each UTLA *i* we found the closest UTLA which was at least 50km distant, denoted by $i_c$. Let $N_i^j$ be the number of cases in UTLA *i* at week *j*, then the expected number of cases in UTLA *i* at week $j+1$ is ${\hat{N}}_{i}^{j+1} = \left( N_{i_c}^{j+1} / N_{i_c}^{j}\right) \cdot N_{i}^{j}$. We refer to the difference, $\delta _i^{j+1} = \left( N_{i}^{j+1} - {\hat{N}}_{i}^{j+1}\right)$, as the case count innovation (similar to Kalman filtering), i.e., the difference between the predicted and measured values. The standard deviation of $\delta _i^{j+1}$ across all UTLAs at week $j+1$ is computed, and abnormal rises in case numbers are defined by rises greater than or equal to two standard deviations.

### Analysis

Analysis was conducted at a weekly resolution, beginning on Mondays of each week, starting on March 4th, 2020. At each week *w* we found for each UTLA *i* and keyword *k* a set of *N* control UTLAs such that $F_{ik}^w$ could be predicted from $\{F_{jk}^w\}_{j=1}^{N}$. To do so, a greedy procedure was followed for each UTLA *i*:

1. Find a UTLA which is at least 50km distant from the *i*-th UTLA for which the linear function $\varvec{F}_{i}^w \approx \omega _1 \varvec{F}_{j}^w$ where $\varvec{F}_i^w$ is a vector of keywords *k*, $k=1, 2, \ldots , 25$. We seek a mapping of the symptom rates at *j* to the symptom rates at *i* which reaches the the highest coefficient of determination ($R^2$). $\varvec{F}_{i}^w \approx \omega _1 \varvec{F}_{j}^w + C$ in the least-squares regression sense. $\omega _1$ is the coefficient of the linear function and *C* is an intercept term.
2. Repeat **(1)**, adding at each time another area that maximally increases $R^2$ when added to the previously established set of areas. That is, at iteration *iter*, find the UTLA which, if added to the previously found UTLAs, minimizes the MSE of the function: $\varvec{F}_{i}^w \approx \omega _1 \varvec{F}_{j_1}^{w} + \cdots + \omega _{iter} \varvec{F}_{j_{iter}}^{w} + C$. Note that the values of $\omega$ and *C* are recomputed at each iteration.

The linear function *f* was optimized for a least squares fit, with an intercept term.

The result of this procedure is a linear function which predicts the symptom rate for each UTLA given the symptom rates at *N* other UTLAs at week *w*. We denote this prediction as $\varvec{{\hat{F}}}_{i}^w = f(\varvec{F}_{jk}^w)$.

We used $N=5$ after observing the changes in $R^2$ as a function of the number of UTLAs (see Supplementary Materials Figures A1).

The function is applied at week $(w+1)$ to each UTLA, and the difference between the estimated and actual symptom rate for each symptom is calculated: $d_{ik} = F_{ik}^{w+1} - {\hat{F}}_{ik}^{w+1}$. We refer to this difference as the **UTLA outlier measure** for symptom *k*.

To facilitate comparison between the differences across keywords, $d_{ik}$, the values of $d_{ik}$ are normalized to zero mean and unit variance (standardized) for each keyword across all UTLAs.

The threshold at which a UTLA should be alerted can be set in a number of ways. In our work with UKHSA, we reported UTLAs where the value of the UTLA outlier measure, $d_{ik}$, exceeded the 95-th percentile threshold of values, computed for all UTLAs with sufficient data and all 23 symptoms excluding ”cough” and ”fever”, similar to the procedure used in the False Detection Ratio test^25^.

### Demographic comparisons

Demographic characteristics of UTLAs were collected from the UK Office of National Statistics (ONS), and include population density^26^, male and female life expectancy and healthy life expectancy^27^, male to female ratio, and the percentage of the population under the age of 15^28^.

Association between demographic characteristics of UTLAs and the likelihood that they would be incorrectly identified as having abnormally high UTLA outlier measure values was estimated using a logistic regression model.

### Ethics approval

This study was approved by the Institutional Review Board of Microsoft.

## Supplementary Information

Supplementary Information.

## Data Availability

Bing data similar to the ones reported here are available online at https://github.com/microsoft/Bing-COVID-19-Data.
