# Supplementary material for: Providing early indication of regional anomalies in COVID-19 case counts in England using search engine queries
Source: Sci Rep. 2022 Feb 11;12:2373. doi: 10.1038/s41598-022-06340-2 (PMC8837788; doi:10.1038/s41598-022-06340-2)
Supplement: Supplementary file 1 — Supplementary Information. [file 41598_2022_6340_MOESM1_ESM.pdf]

1 Appendix

Figure A1 shows the improvement in model fit (R^2) as more areas are added to $\boldsymbol{F}_i^w$. As the model shows, improvement continues, but the marginal gain decreases with the number of areas, as expected.

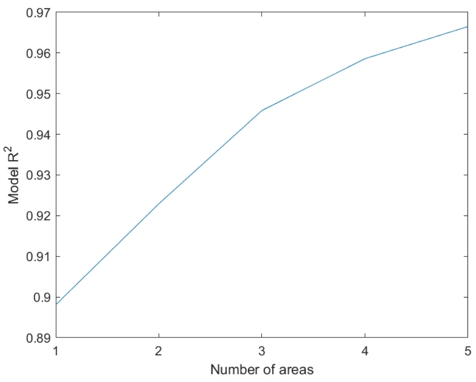

Figure A1. Average R^2 values of the model for $\boldsymbol{F}_i^w$ as the number of areas increases.

Figure A2 shows the ROC for "cough" at a lag of 8 days during the first wave of the pandemic.

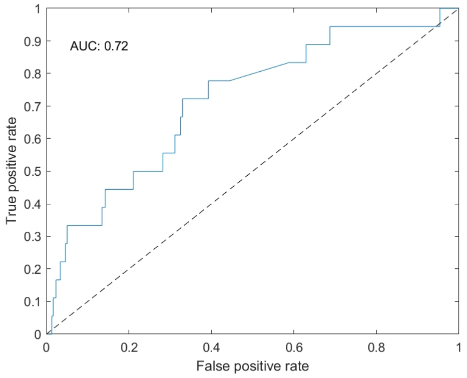

Figure A2. Receiver Operating Curve (ROC) for "cough" at a lag of 8 days.
